# Reframing continuity of care: A family medicine specialist's perspective on occupational therapy for neurodevelopmental disorders

**DOI:** 10.51866/mol.26-00008

**Published:** 2026-08-27

**Authors:** Shahira Ismail

**Affiliations:** 1 Klinik Kesihatan Segamat, Jalan Muar, Segamat, Johor, Malaysia.

**Keywords:** Occupational therapy, Primary health care, Neurodevelopmental disorders, Continuity of patient care, Early intervention

Children with neurodevelopmental disorders (NDDs) commonly present in primary care with developmental delays, behavioural concerns and learning difficulties. My role as a family medicine specialist traditionally centres on early identification, diagnosis, caregiver counselling and longitudinal follow-up. Despite regular clinical encounters, many children continue to experience challenges with functional independence, participation, and daily living skills. This reflects a gap between clinical management and meaningful real-world outcomes.

My recent attachment to the occupational therapy (OT) unit provided valuable insights into how to address this gap. I was exposed to the assessment and management of children with conditions such as autism spectrum disorder, attention-deficit/hyperactivity disorder, and global developmental delay. Unlike the clinic-based model of care, OT emphasises functional outcomes, focusing on children’s ability to perform activities of daily living, engage in play and participate in school and social environments.

OT assessments extend beyond diagnostic categorisation to include sensory processing, motor coordination, adaptive behaviour and environmental influences. Interventions are highly individualised, incorporating play-based strategies, sensory integration techniques and structured caregiver involvement. This ensures that therapeutic gains are reinforced within the children’s natural environment, promoting sustained functional development.

This experience prompted a shift in my understanding of continuity of care. In primary care, continuity is often defined by regular follow-up and sustained doctor–patient relationships. However, in the context of NDDs, continuity must also encompass ongoing functional progress in children’s daily life. OT plays a central role in achieving meaningful functional outcomes by translating clinical goals into real-world participation and independence. In this way, OT complements primary care by extending care beyond clinic-based encounters into children’s home and community.

The attachment also highlighted the importance of a biopsychosocial and family-centred approach. Occupational therapists actively engage caregivers and consider family dynamics, environmental factors children’s developmental context when planning interventions. This operationalises holistic care in a structured and practical manner, reinforcing the role of family physicians as coordinators within multidisciplinary teams.

Early intervention emerged as another critical theme. Timely referral to OT can optimise developmental outcomes by leveraging neuroplasticity during early childhood. Family medicine specialists are well-positioned to identify early signs of developmental delay and initiate appropriate referrals, ensuring that children receive comprehensive and timely care.

However, challenges remain in integrating OT into primary care. Limited access, long waiting times and variable awareness of OT services may delay intervention and affect outcomes. Addressing these barriers requires improved referral pathways, stronger interprofessional collaboration and greater integration of allied health services within primary care settings.

In conclusion, OT plays a significant role in enhancing the primary care management of NDD. By focusing on functional outcomes and real-world participation, it bridges the gap between clinical care and everyday functioning, ultimately strengthening continuity of care and improving longterm developmental outcomes.

